# The Role of Dll4/Notch Signaling in Normal and Pathological Ocular Angiogenesis: Dll4 Controls Blood Vessel Sprouting and Vessel Remodeling in Normal and Pathological Conditions

**DOI:** 10.1155/2018/3565292

**Published:** 2018-07-05

**Authors:** Ivan Lobov, Natalia Mikhailova

**Affiliations:** Cell Technologies Center, Institute of Cytology of the Russian Academy of Sciences, Tikhoretsky Ave. 4, Saint Petersburg 194064, Russia

## Abstract

**Background:**

Retina is the highest oxygen-demanding and vascularized tissue in the body. Retinal development and function require proper vascularization and blood vessel function and integrity. Dll4 is most prominently expressed in the endothelium of angiogenic blood vessels and in quiescent arteries and capillaries in all tissues and organs of the mammalian species, and it is the key regulator of blood vessel sprouting.

**Results:**

Dll4 is a transmembrane protein that acts as a ligand for Notch receptors 1 and 4. Genetic deletion of Dll4 causes severe abnormalities in embryonic and postnatal vascular development. Deletion of even a single Dll4 allele results in almost complete embryonic lethality due to severe vascular abnormalities, the phenomenon called haploinsufficiency indicating the critical role of Dll4/Notch in vascular development. Dll4/Notch pathway interplays at multiple levels with other signaling pathways including VEGF, Wnt/Fzd, and genes controlling vascular toning. Multiple studies of the effects of Dll4 inhibition were performed in the developing retina to elucidate the key functions of Dll4 in normal and pathological angiogenesis. Several genetic approaches and therapeutic molecules were tested to evaluate the biological and therapeutic effects of acute and prolonged Dll4 inhibition in the eye and oncology.

**Conclusions:**

All current studies demonstrated that Dll4 controls blood vessel sprouting, growth, and remodeling in normal and pathological conditions as well as arterial-venous differentiation. Genetic and therapeutic Dll4 modulation studies show that Dll4 inhibition can promote blood vessel sprouting and might be useful to stimulate vessel growth in the ischemic retina and Dll4 is the key modulator of the postangiogenic vascular remodeling that ultimately defines vascular patterning.

## 1. Dll4/Notch Signaling Pathway in Angiogenesis

Vascular system is the first functional system in development, and retina is the highest oxygen-demanding tissue in the body [1]. Angiogenesis is the physiological process through which new blood vessels form from preexisting vessels [1, 2]. In precise usage, this is distinct from vasculogenesis, which is the *de novo* formation of endothelial cells from mesoderm cell precursors, and from neovascularization [3]. The first vessels in the developing embryo form through vasculogenesis, after which angiogenesis is responsible for most, if not all, blood vessel growth during development and in disease [4].

**Figure 1:**
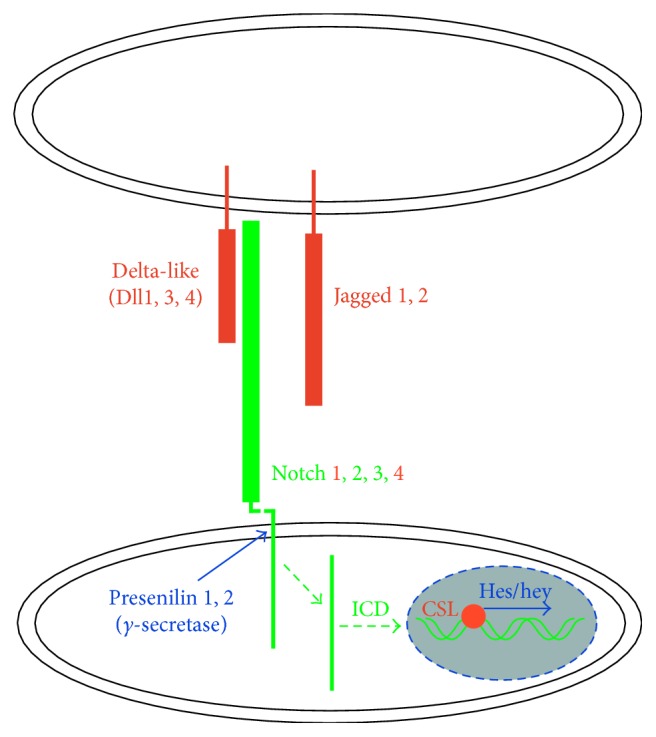
Dll4/Notch signaling in the endothelial cells. Notch receptor activation by Dll and Jagged ligands expressed by the neighboring cells leads to the cleavage of the intracellular domain (NICD) of the receptor, its translocation into the nucleus, and blockade of CSL that allows the transcription activation of the downstream genes by transcription factors of Hes and Hey families [14].

Notch signaling pathways are evolutionary conserved and control cell-fate determination and differentiation in multiple tissues and cell types [5–7], including the vascular system [8]. Signaling through Notch 1 and Notch 4 receptors has been implicated in regulating angiogenesis and vascular differentiation during development and in diverse pathological conditions [9–11]. There are several Notch ligands Dll1, 2, 4, and Jagged 1, 2 acting through Notch receptors 1–4. Notch activation leads to the cleavage of the intracellular Notch domain NICD by presenilin/*γ*-secretase and its nuclear translocation, where it interacts with a complex composed of RBP-Jk/CSL and mastermind-like proteins (MAML) [12] to induce the transcription of target genes that regulate cell-fate determination and other events (Figure 1), for example, cell migration and proliferation in the normal vasculature and malignancy [13].

Dll4 is the only Notch ligand expressed predominantly by the vascular endothelium [15–17], and mice lacking even a single Dll4 allele exhibit multiple severe vascular abnormalities, including defective arteriogenesis, resulting in embryonic lethality in most mouse strains. For other genes associated with angiogenesis, lethal haploinsufficiency has been previously observed only in *VEGF-A* mutants [18, 19], further emphasizing the critical nature of Dll4 in vascular development. Vascular endothelial growth factor (VEGF) is a key modulator of angiogenesis during normal development and in pathology promoting vascular endothelial cell proliferation and blood vessel outgrowth as well as vessel wall permeability [20, 21]. The VEGF-A and Dll4/Notch signaling pathways interplay at several levels in vascular development and pathology [22, 23]; for example, VEGF-A robustly induces Dll4 expression in the cell culture and in the developing vasculature, while Jagged 1 expression in the vascular endothelium activates Notch signaling in vascular smooth muscle cells/pericytes and maintains stability and function of adult vasculature [24]. Dll4/Notch pathway interplays at different levels with other signaling pathways including Wnt/Fzd [25]. Other signaling pathways that also involved in retinal angiogenesis in normal and pathological conditions include angiopoietin/Tie2 [26, 27] and apelin/APJ [28] signaling pathways; however, they are predominantly involved in vascular proliferation and blood vessel growth rather than control of blood vessel sprouting.

## 2. Dll4 Expression in the Normal Retinal Blood Vessel Development

**Figure 2:**
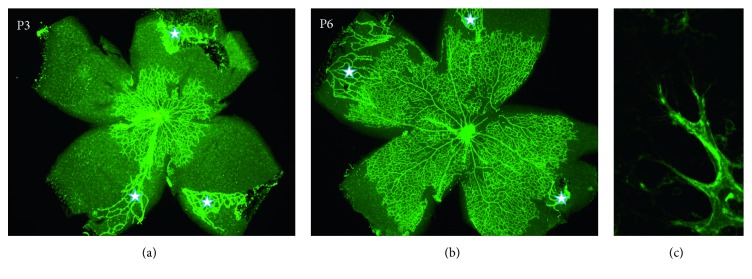
Retinal blood vessel development in the mouse eye. Retinal blood vessels start to grow at the postnatal day 1 (P1) from the optic nerve head in the center of the retina and extend toward periphery and achieve the retinal periphery by P8. At P3, developing vasculature covers approximately 30% of the retina (a), but by P6, about 70–80% of the retina is covered by the developing vessels (b). Angiogenic sprouts at the growing front of the developing vasculature have multiple filopodia extending from the tip cells (c). GS lectin staining: 2x magnification (a, b); 10x magnification (c). At the same time, there is an ongoing regression of the hyaloid vessels [30, 31]. Remaining hyaloid blood vessels are labelled by asterisks.

Mouse postnatal retinal vasculature develops after birth in a highly stereotypic manner during which time it is accessible to experimental manipulations that makes it ideal for the studies of pro- and antiangiogenic molecules and the effects of genetic mutations on normal and pathological angiogenesis (Figure 2) [29]. In the mouse retina, superficial retinal blood vessels start growing rapidly from the optic nerve head toward retinal periphery at birth, and by postnatal day 3 (P3), approximately 30% of the retina is covered by vascular network (Figure 2(a)), and by P6, approximately 70% is covered by vessels (Figure 2(b)), and retinal vascularization is completed by P8. Growing blood vessels are guided by highly specialized “tip” cells extending multiple filopodia.

**Figure 3:**
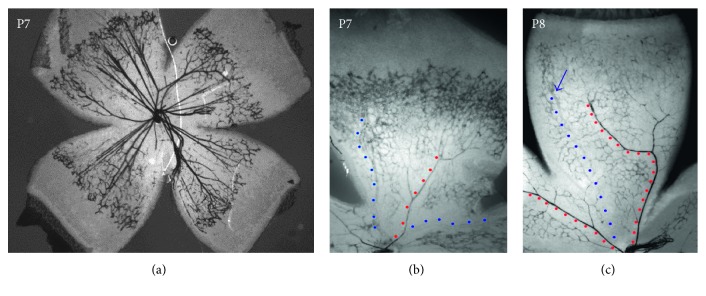
Dll4/lacZ expression in the developing mouse retina of Dll4^+/−^ heterogeneous mice at P7 (a, b) and P8 (c). Dll4 is predominantly expressed in the capillaries at the growing front of the developing retinal vasculature and differentiated arteries (red dots) but not veins (blue dots) at P7 (b) and at P8 (c). X-gal staining: 2x (a) and 4x (b, c) magnification [33].

In the developing superficial retinal vasculature and all other forming vascular beds, Dll4 specifically expressed at the tips of the sprouting blood vessels (Figure 3). Under those conditions, VEGF induces Dll4 expression in the specialized “tip” cells that guide migration of the growing capillaries and “stalk” cells that are primarily responsible for proliferation and thus growth of new angiogenic blood vessels [32], where it acts as a negative regulator of VEGF action by suppressing further tip cell formation and proliferation of the stalk cells [33, 34]. Dll4 expression determines “tip” cell identity by regulating expression of a number of cell surface receptors including VEGFR-2, VEGFR-3, NRP1, CD34, Unc5B, PDGF-BB, and Kcne3 ([35–37]; unpublished data IBL). “Tip” cell localized at the forefront of vessel branches shows highly polarized nature and numerous filopodia probing the environment, while migrating toward an angiogenic stimulus. They do not form a lumen and proliferate minimally.

However, Dll4 is expressed not only in “tip” and stalk cells during active angiogenesis, it is also expressed at particularly high levels in the vascular endothelium of mature arteries and arterioles, and to a lesser extent in capillaries, but not veins [15, 33] (Figure 3). Moreover, in contrast to Dll4 expression in angiogenic vessels, the expression of Dll4 in postangiogenic vessels does not appear to be regulated by VEGF-A [33]. VEGF inhibition with VEGF Trap (aflibercept) effectively blocked Dll4/LacZ expression at the growing front of the developing retinal vasculature, while Dll4/LacZ expression remained unaffected in the differentiated arteries, indicating that there are two different mechanisms regulating Dll4 expressing in the vasculature [33]. Endothelial Notch signaling most likely through Dll4 is also involved in maintaining vascular patterns in the adult tissues [38].

## 3. Effects of Dll4 Inhibition on Normal Angiogenesis in the Developing Retina

**Figure 4:**
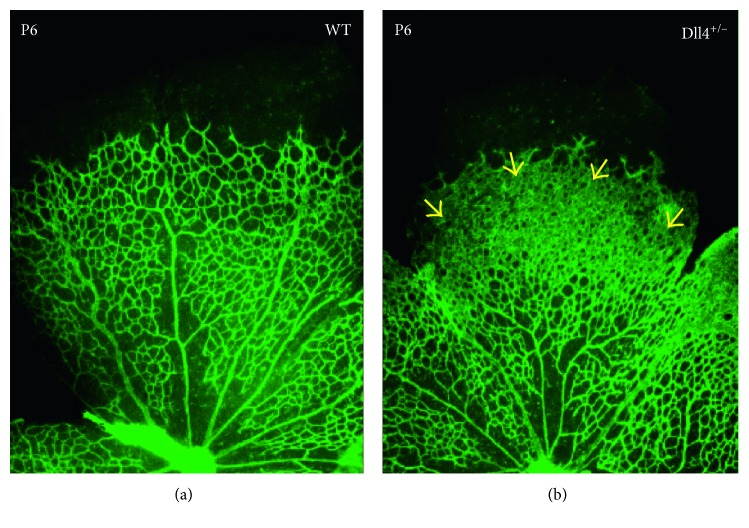
Retinal vascular development in normal (WT) (a) and Dll4-deficient heterozygous mice (Dll4^+/−^) (b) at P6. Heterozygous Dll4 deletion causes severe developmental retinal vascular abnormalities leading to the increased blood vessel sprouting and formation of a syncytium-like vascular plexus (yellow arrowheads). 4x magnification. GS lectin staining. [33].

VEGF-A is the main growth factor controlling endothelial cell proliferation and blood vessel outgrowth [21, 39], while Dll4 negatively controls blood vessel sprouting and new tip cell formation [40]. Notably, in all species, and under all normal and pathological angiogenic conditions evaluated to date, Dll4/Notch inhibition stimulates vascular sprouting and endothelial cell proliferation [41, 42]. During normal retinal development, inhibition of the Dll4/Notch pathway dramatically increases angiogenic sprouting, resulting in the formation of an abnormally dense primary capillary plexus, in which blood vessels fuse to form a syncytium [33, 40, 43]. (Figure 4); yet in these settings, blood vessels are fully perfused and functional [33]. Intriguingly, in tumors, pharmacological inhibition of Dll4 promotes hyperproliferation of nonperfused blood vessels that is increasing tissue ischemia and suppresses tumor growth [34, 44]. That discrepancy is most likely due to excessive and chaotic tumor vasculature growth when additional blood vessel proliferation leads to even poorer tissue oxygenation.

It was also found that the degree of vascular abnormalities and tissue side effects depend on the potency of Dll4 inhibition, namely, on the dose and reagent affinity ([45, 46]; unpublished data IBL).

## 4. Effects of Dll4 Inhibition on Pathological Angiogenesis and Retinal Neovascularization in the Eye

**Figure 5:**
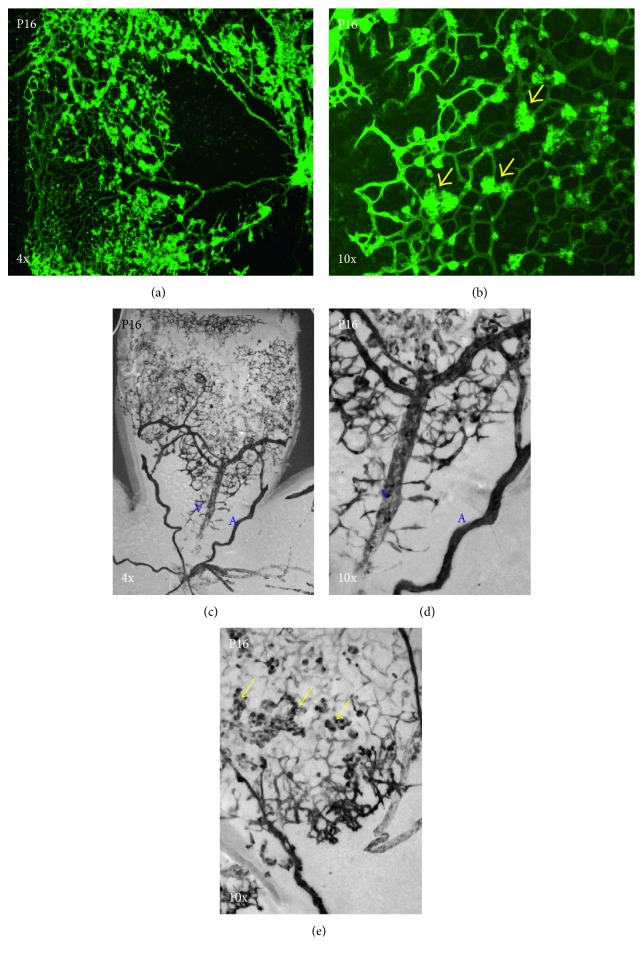
Pathological neovascularization in OIR (oxygen-induced retinopathy model) at P16 and Dll4 expression in OIR at P16. Dll4 is expressed in the capillaries, arteries (a), and pathological neovascular tufts (yellow arrows) in OIR in pathological angiogenesis but at a lower level in veins (V). X-gal staining: 4x (a, c) and 10x (b, d, e) magnification. Yellow arrows indicate pathological neovascular tufts.

In the oxygen-induced model of ischemic retinopathy (OIR) [47] that mimics ROP (retinopathy of prematurity), an infant ocular disorder that develops in prematurely born babies is exposed to high oxygen [48]. In the OIR model, neonatal mouse pups are exposed to high oxygen levels that lead to massive vasoobliteration of the immature retinal vasculature and upon return to normal room air that initiates excessive pathological angiogeneses and formation of nonperfused neovascular tufts protruding into vitreous (Figures 5(a) and 5(b)). In this model, Dll4 is broadly expressed as in the normal development in the arteries and capillaries, but it is also upregulated in veins and most prominently in neovascular tufts (Figures 5(c)–5(e)).

**Figure 6:**
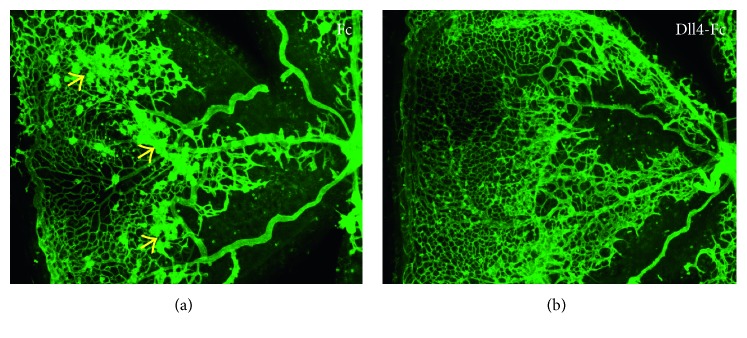
Pharmacological inhibition of Dll4 in OIR using soluble Dll4-Fc fusion protein (b) ameliorates pathological retinal neovascularization and improves normal retinal revascularization in the OIR model [33]. Yellow arrows indicate pathological neovascular tufts.

To evaluate the therapeutic potential of Dll4 inhibition in ocular vascular diseases, Dll4-Fc chimeric recombinant protein (a soluble Dll4/Notch inhibitor) and an anti-Dll4-blocking antibody were utilized in the murine model of OIR in multiple settings. In OIR, Dll4/Notch inhibition results in a more rapid revascularization of the ischemic portions of the retina [33], while surprisingly ameliorating pathological angiogenesis (neovascularization and neovascular tufts formation) (Figure 6). It was suggested that the ability of Dll4/Notch inhibitors to ameliorate pathological angiogenesis is based on their ability to promote normal angiogenesis and thus ameliorate the tissue ischemia by further reducing the levels of angiogenic factors responsible for pathological blood vessel growth.

There are several severe retinal vascular disorders besides ROP causing blindness that are characterized by blood vessel loss at the initial stages and severe retinal ischemia, and as a result, excessive pathological neovascularization on the terminal phases that include diabetic retinopathy (DR) [49] that primarily affects retinal vasculature and wet age-related macula degeneration (AMD) [50] that is caused by excessive growth of choroidal blood vessel into retina. Dll4/Notch pathway is actively involved in the progression of these diseases [7]. There is a possibility that Dll4/Notch pathway modulation in these diseases especially at the early stages can also prevent or significantly ameliorate disease progression.

## 5. Effects of Dll4 Inhibition on Normal Physiological Blood Vessel Remodeling and Blood Vessel Regression in the Retina

Although the role of Dll4 in sprouting angiogenesis has been extensively studied, until recently, very little was known about the function of the Dll4/Notch pathway in postangiogenic and mature blood vessels, where Dll4 is expressed in the arteries.

Genetic or pharmacological modulation of Dll4/Notch signaling affects postangiogenic blood vessel remodeling and regression, a process that plays a critical role in determining the patterning and density of blood vessels in mature tissues. Specifically, constitutive or conditional genetic deletion of Dll4 in ROSA26–CreERT2+/−; Dll4^COIN/+^mice or pharmacological inhibition of Dll4/Notch signaling using Dll4-Fc inhibited the normal developmental pruning of capillaries in the developing retinal vasculature, as well as blood vessel regression following the acute exposure to hyperoxia in OIR [51].

Genetic deletion of a negative feedback regulator of the Notch pathway, Notch-regulated ankryrin repeat protein (Nrarp) [52], results in an opposite Notch gain-of-function phenotype leading to exaggerated blood vessel regression. In both normal development and the OIR model, Nrarp-knockout mice exhibited significant reductions in retinal vessel density compared to wild-type littermates [25]. Moreover, in OIR-treated mice, Nrarp-null mice showed increased vasoobliteration at P12 and at P17 as well as increased pathological neovascularization. Mice heterozygous for Nrarp deletion exhibited an intermediate phenotype. Thus, Nrarp deficiency produced effects opposite to those observed following genetic or pharmacological inhibition of the Dll4 [51].

**Figure 7:**
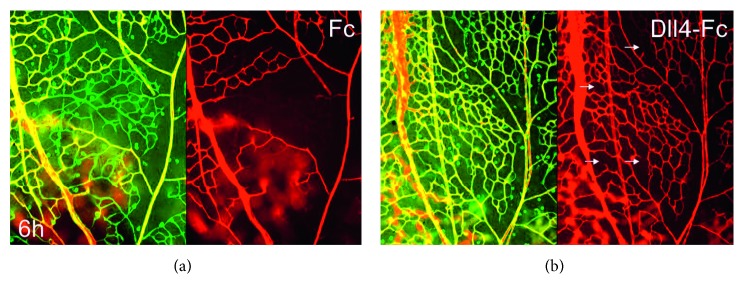
Pharmacological inhibition of Dll4 in acute hyperoxia (6 hours, 75% O2) using soluble Dll4-Fc fusion protein (b) ameliorates pathological retinal capillary nonperfusion compared to Fc control (a). GS lectin-positive and tomato lectin-negative capillaries are transiently nonperfused. Small white arrows indicate nonperfused capillary segments (b). Nonperfused capillaries rapidly regress within 24 hours. Note large nonperfused areas in the control retinas and just a few individual nonperfused segments in Dll4-Fc-treated retinas. Green: GS lectin staining; red: tomato lectin perfusion [51].

In OIR, blood vessel regression is preceded by the excessive vasoconstriction of the immature capillaries and loss of blood flow, followed by rapid synchronous apoptosis in the capillaries [51]. In some earlier studies, synchronous apoptosis was also shown during regression phase in the pupillary membrane of the rat eye and hyaloid blood vessels [53–55]. Blood flow can be improved and maintained by Dll4/Notch inhibition using Dll4-Fc (Figure 7) [51]. This effect was associated with induction of the genes that encode vasodilatory peptides (e.g., adrenomedullin), while suppressing the expression of genes involved in vasoconstriction (e.g., angiotensinogen). The data indicate that Dll4/Notch pathway regulates blood vessel remodeling and regression by rapidly fine-tuning the expression levels of a distinct subset of vasoactive genes.

Effects of Dll4/Notch signaling modulation were largely distinct from changes produced by modulation of VEGF-A activity, at both morphological and gene expression levels, indicating that these two pathways independently regulate postangiogenic blood vessel remodeling and homeostasis [51].

From the therapeutic perspectives, importantly, attenuation of Dll4/Notch signaling maintained blood flow and prevented capillary regression without inducing the gross morphological changes in the postangiogenic vasculature that were evident following administration of VEGF-A [51]. Noteworthy, VEGF-A produced very rapid gross vascular abnormalities leading to formation of extremely dilated baggy veins and capillaries as well as surprising additional capillary pruning near arteries. The ability of Dll4/Notch signaling to modulate blood flow and subsequent vascular regression via angiotensin and adrenomedullin pathways represents a new mechanism by which this pathway modulates vascular development and remodeling [51]. Thus in this study, it was demonstrated, for the first time, a nonangiogenic role of Dll4/Notch in the remodeling of maturing vasculature, which in contrast to its role in sprouting angiogenesis appears to be largely independent of VEGF signaling.

Conditional genetic deletion of another transcription factor, negative-feedback regulator of Notch signaling pathway, recombination signal-binding protein Jkappa (RBP-J) in the adult mice, strikingly induced spontaneous angiogenesis in the multiple tissues, including retina and cornea [56]. That data also indicate that Notch signaling is critical in the established mature vasculature to maintain blood vessel integrity and function.

Recent data also suggest that Dll4/Notch signaling is involved in arteriogenic differentiation that further limits angiogenic sprouting and maintains arterial endothelium in nonproliferative state [57, 58].

## 6. Conclusions

Dll4/Notch signaling pathway modulates angiogenic sprouting and blood vessel growth, and Dll4/Notch inhibition can promote therapeutic angiogenesis in the ischemic tissues and at the same time over exuberate pathological angiogenesis in solid tumors showing anticancer effect. Dll4/Notch inhibition can also maintain capillary perfusion and prevent pathological blood vessel regression, for example, induced by exposure to hyperoxia. A number of diseases, such as retinal ischemic disorders, cerebral, and cardiac ischemia, are characterized by vascular insufficiency resulting in reduced tissue perfusion [59]. In these settings, attenuating vessel loss, improving flow in existing vessels, and promoting angiogenesis might have beneficial effects. Taken together with previous observations, available data suggest that Dll4/Notch inhibition can promote productive angiogenesis and vessel regrowth in the ischemic retina tissue [30]; all available data suggest that Dll4/Notch inhibitors might be useful for ameliorating diverse ischemic conditions and disorders characterized by progressive loss of blood vessels and insufficient compensatory angiogenesis in and beyond the eye. Development of new antibodies and soluble ligands modulating Dll4/Notch signaling will allow preventing blood vessel loss and promote therapeutic productive angiogenesis in the retina and potentially other tissues and organs.

## Abbreviations

Dll4: Delta-like ligand 4
DR: Diabetic retinopathy
Fc: Constant Fc region of human IgG
GS lectin: *Griffonia simplicifolia* lectin I
Nrarp: Notch-regulated ankyrin repeat protein
OIR: Oxygen-induced retinopathy
VEGF-A: Vascular endothelial growth factor A
wet AMD: Wet form of age-related macular degeneration
WT: Wild type.
